# Supplementary figures and images for: Endocytic coelomocytes are required for lifespan extension by axenic dietary restriction
Source: PLoS One. 2023 Jun 27;18(6):e0287933. doi: 10.1371/journal.pone.0287933 (PMC10298762; doi:10.1371/journal.pone.0287933)

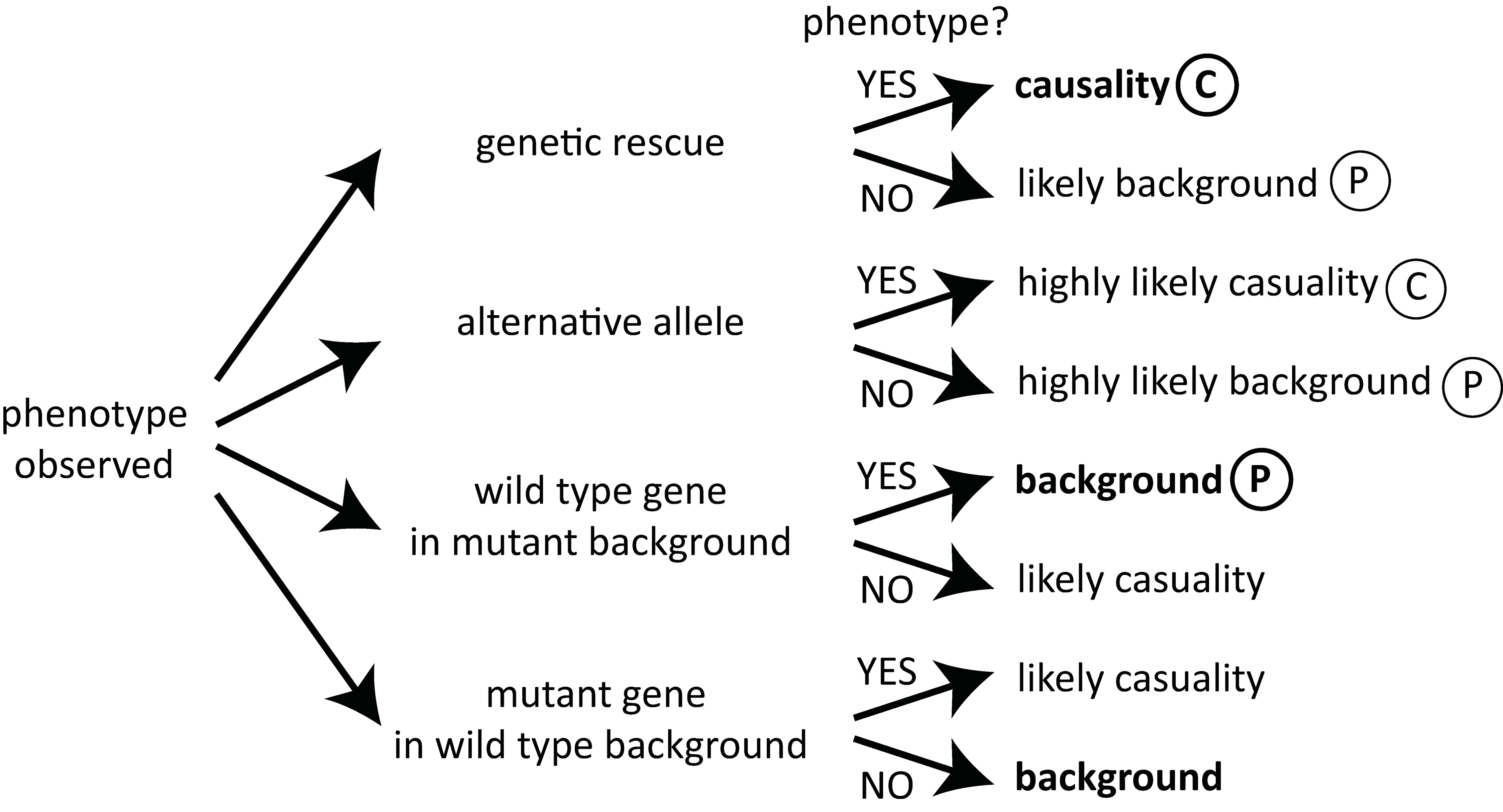

Supplement: S1 Fig — When a phenotype is observed in a genetic knockout mutant, different approaches can be used to further prove causality, as shown here. Outcomes in bold are those that are conclusive, others form strong indications. In our experiments, an ADR lifespan phenotype was initially observed for cup-4(ok837) and pmk-1(km25). Further steps taken and their outcome are marked with C (cup-4) and P (pmk-1). (TIF) [file pone.0287933.s001.tif]

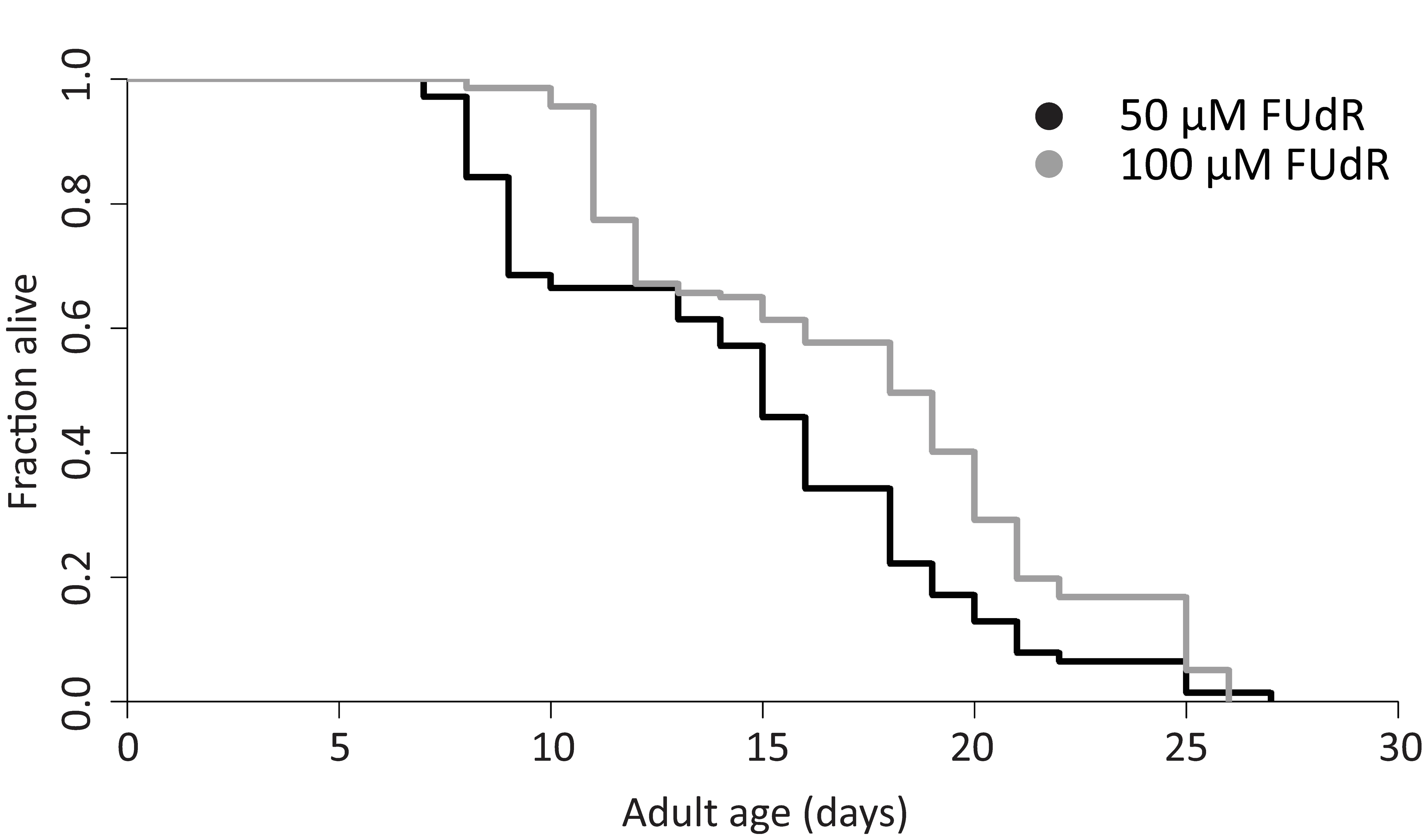

Supplement: S2 Fig — Lifespan in FF conditions of dbl-1(nk3) worms at a concentration of either 50 μM or 100 μM FUdR. At a lower FUdR concentration, dbl-1(nk3) mutants are significantly shorter lived (p = 1.1E-04) than at the higher concentration. Mean lifespans, sample numbers and statistical significance are shown in S1 Table. (TIF) [file pone.0287933.s002.tif]

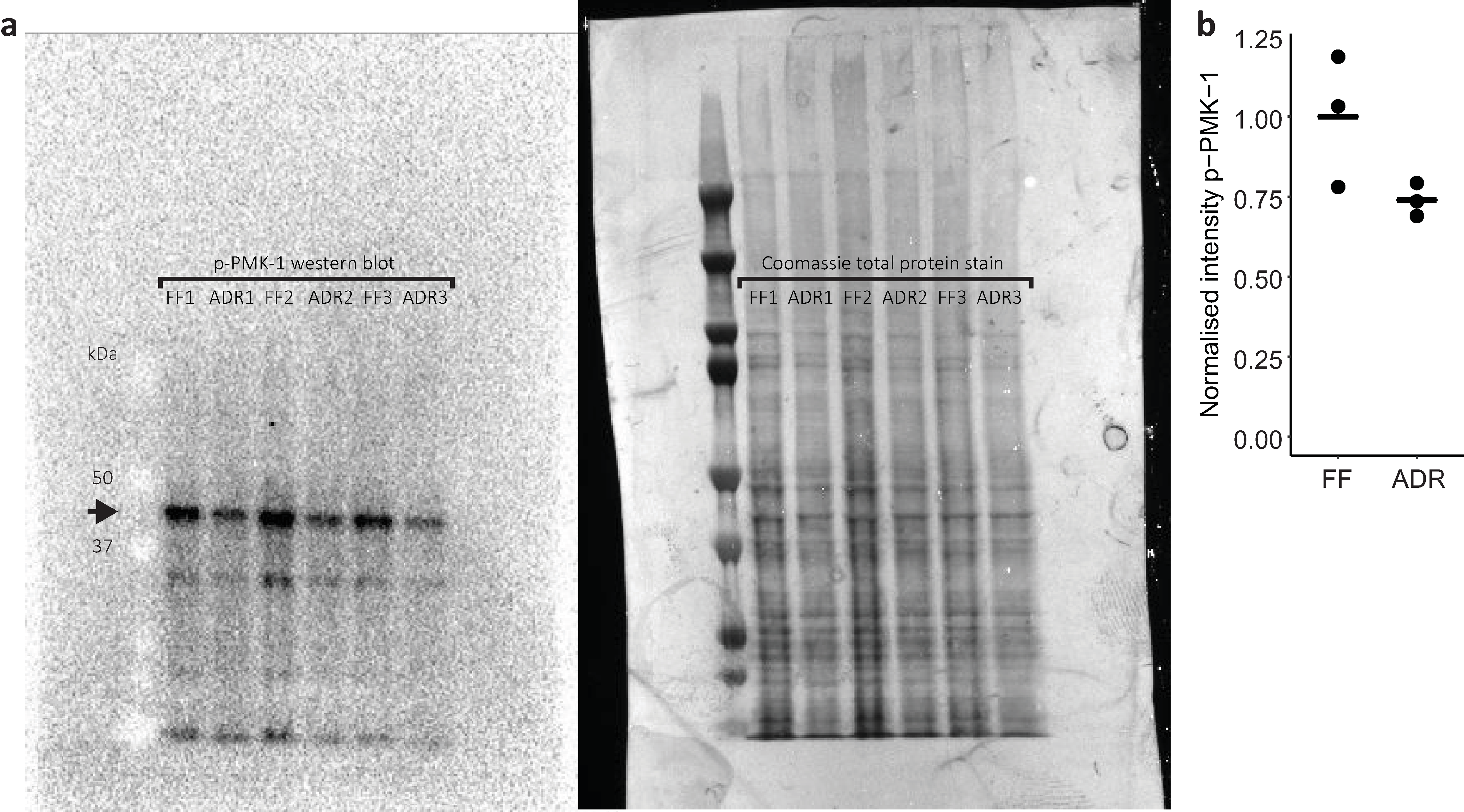

Supplement: S3 Fig — A. Uncropped western blot and Coomassie total protein stain of three biological replicates under fully fed (FF) and axenic dietary restriction (ADR) conditions. phospho-PMK-1 (p-PMK-1) band is marked by an arrow. B. Quantification of p-PMK-1 levels as measured by Western blot and normalized to total protein. PMK-1 activity was not increased under ADR, as no significant difference was observed based on an unpaired two-samples t-test, with a possible trend to lower activity levels under ADR. (TIF) [file pone.0287933.s003.tif]

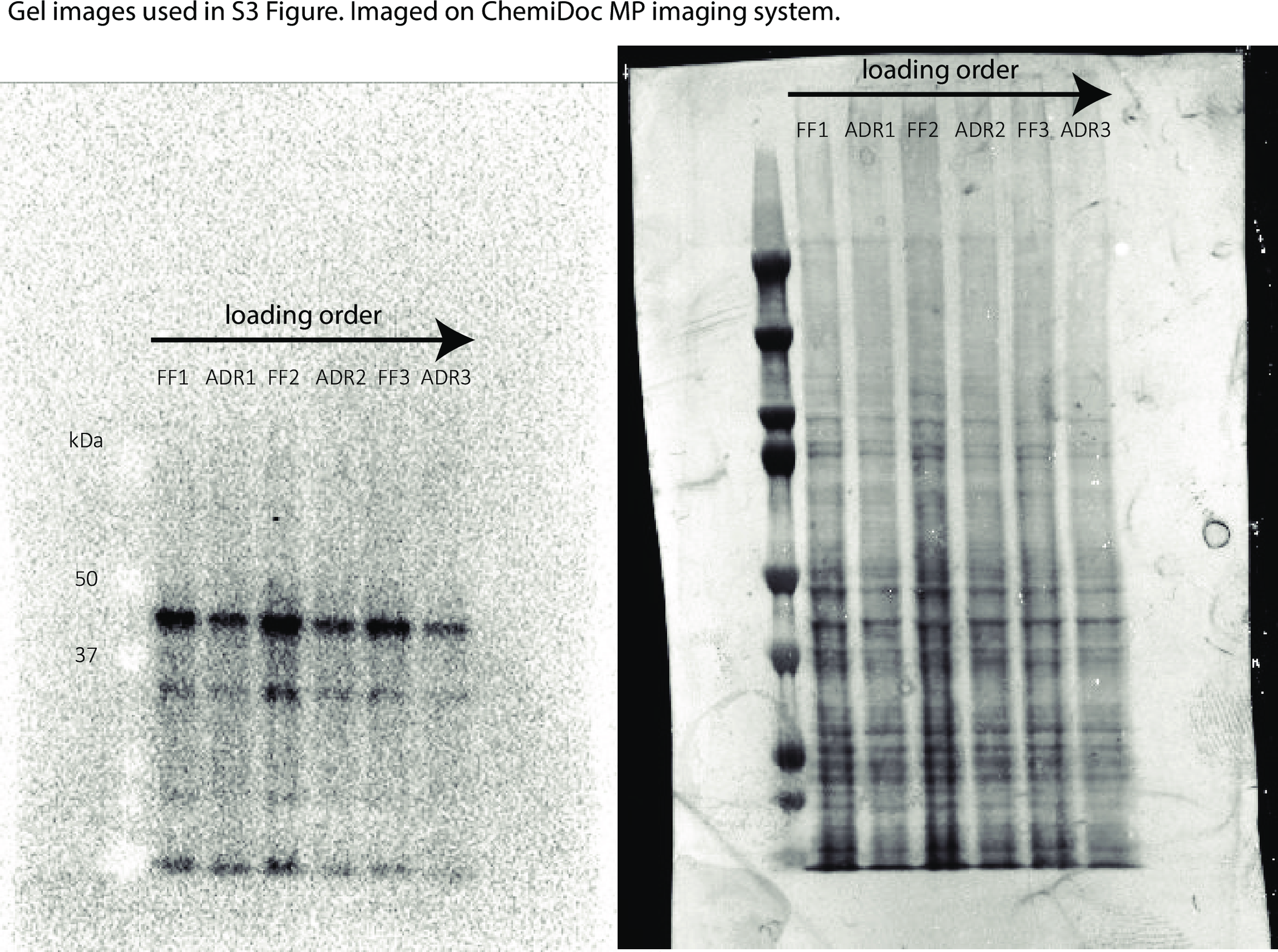

Supplement: S1 Raw images — (TIF) [file pone.0287933.s006.tif]
