# Supplementary material for: Endocytic coelomocytes are required for lifespan extension by axenic dietary restriction
Source: PLoS One. 2023 Jun 27;18(6):e0287933. doi: 10.1371/journal.pone.0287933 (PMC10298762; doi:10.1371/journal.pone.0287933)
Supplement: S1 Methods — (PDF) [file pone.0287933.s005.pdf]

***p38-MAPK activity assay***

p38-MAPK activity was determined via Western blot. Worms were grown as described under *Fluorescence microscopy*, in fully fed or axenic conditions. On day 2 of adulthood, worms were pipetted onto unseeded NGM plates and 50 worms were picked into 5 µL S basal and snap-frozen. XT sample buffer (Bio-Rad) and XT reducing agent (Bio-Rad) were added and the sample was vortexed at 70 °C for 15 min, then heated to 95 °C for 5 min. After a short spin-down, samples were loaded onto a Criterion XT Bis-Tris 4-12% Precast Gel (Bio-Rad) alongside a Precision Plus Protein Dual Xtra Prestained Standard (Bio-Rad) and run at 200 V in MOPS buffer (Bio-Rad) in a Criterion tank (Bio-Rad). Proteins were transferred onto a PVDF membrane using the Trans-Blot Turbo Transfer System (Bio-Rad). The membrane was blocked for 2 hours in Tris containing 5% BSA (Sigma), and then incubated overnight, shaking at 4 °C in Tris Saline containing 2.5% BSA and 1:200 Phospho-p38 MAPK (Thr180/Tyr182) antibody (Cell Signaling, 9211S). The following day, the membrane was washed 1x 3 min and 2x 7 min in Tris Saline, and then incubated for 45 min in Tris Saline containing 1:50 000 Polyclonal Goat Anti-Rabbit Immunoglobulins HRP (DAKO). This was followed by 5x 7 min washes in Tris Saline and 1x 5 min wash in Tris, before incubation with Supersignal West Dura (Thermo Scientific) in the dark and visualisation on a ChemiDoc MP imager (Bio-Rad). Membranes were then washed 3x 5 min in MQ, stained for 10 min in Coomassie Brilliant Blue R-250 (Bio-Rad), and destained 2x 10 min with 50% methanol and 7% acetic acid, to determine total protein levels. Image Lab (Bio-Rad) was used to calculate the intensity of the p-PMK-1 band at 43.9 kDa, as well as that of the six clearest bands of total protein. The data was normalised by dividing the p-PMK-1 value by the total protein value, producing a measure for PMK-1 phosphorylation (and thus PMK-1 activity).
