# Supplementary material for: Endocytic coelomocytes are required for lifespan extension by axenic dietary restriction
Source: PLoS One. 2023 Jun 27;18(6):e0287933. doi: 10.1371/journal.pone.0287933 (PMC10298762; doi:10.1371/journal.pone.0287933)
Supplement: Attachment — Submitted filename: Mergan et al.pdf [file pone.0287933.s007.pdf]

Mergan et al.

In this manuscript Mergan et al. demonstrate 1. that CUP-4 acts in coelomocytes to regulate lifespan extension under axenic dietary restriction and 2. that the immune regulator PMK-1 is dispensable for ADR-mediated lifespan extension. The construction and use of CRISPR mutant alleles for *cup-4* and *pmk-1* strengthens the conclusions about the involvement of *cup-4* and the dispensability of *pmk-1* in ADR-mediated longevity. It is important to address how much of the reduction in ADR-mediated lifespan extension observed upon coelomocyte ablation is accounted for by CUP-4, and whether other immune pathways act independently of coelomocytes to regulate lifespan upon ADR. I make some suggestions below for addressing these:

1. Does endogenous CUP-4 rescue completely rescue the reduction in ADR-mediated lifespan extension in coelomocyte-ablated animals?

2. Do other components involved in coelomocyte endocytosis regulate ADR-mediated lifespan extension?

3. (a) The *dbl-1* mutant shows a reduction in ADR-mediated lifespan extension – does this effect persist in a lifespan assay performed without FuDR?
   (b) Are the effects of *dbl-1* and coelomocyte ablation (or *cup-4* loss-of-function) on the extended lifespan under ADR additive, i.e., do DBL-1 and coelomocyte endocytosis act independently to regulate lifespan under ADR? If so, then the paper can be about how coelomocyte endocytosis and innate immune pathways independently regulate lifespan upon ADR.

4. How does CUP-4 regulate lifespan extension in ADR? Does LIPL-5 in coelomocytes or intestinal fat content change upon ADR and do these involve CUP-4 or coelomocyte endocytosis?

5. (minor) Does cup-4 expression in wt background extend wt lifespan?

I also suggest the following text and figure changes for clarity:

1. The authors have included p-values in Figure legends – they can consider also including them in the figures on top of the bar plots or next to the survival curves, at least for some of the comparisons.
2. Page 2, line 36 – consider replacing ‘DAF-16a’ with ‘DAF-16’
3. In Figure 2d, consider replacing ‘wt (backcrossed)’ with’ wt (KU25 outcrossed to N2)’
4. Figure S1 – typo: replace ‘oberved’ with ‘observed’
5. Page 10, lines 166-167; if the authors do not find a link between coelomocyte endocytosis and other immune pathways and instead discover that they act independently (see experiments suggested above) they can consider rephrasing these sentences to ‘Coelomocytes are immune cells which ‘phagocytose’ pseudocoelomic content. We wondered whether other immune pathways are involved in ADR-mediated longevity.
6. Page 9, line 151 – ‘localization’ instead of ‘localisation’
7. Page 10, line 183 – typo: change to ’slightly short-lived (p=1.9E-05) under ADR, but an interaction effect with FUdR is likely’
8. Page 13, line 241 – ‘hypothesized’ instead of ‘hypothesised’
9. Figure 1 – consider rearrange panels? The order of C and D are confusing.
10. For Figure S2, consider referring to Table S1.
