# Supplementary material for: Endocytic coelomocytes are required for lifespan extension by axenic dietary restriction
Source: PLoS One. 2023 Jun 27;18(6):e0287933. doi: 10.1371/journal.pone.0287933 (PMC10298762; doi:10.1371/journal.pone.0287933)
Supplement: Attachment — Submitted filename: Response to Reviewers.docx [file pone.0287933.s008.docx]

**In-house Editorial Comments**

After careful reading of the manuscript and the reviewer comments, it is clear that major revisions are required. Reviewer 1 points out that more background on the dietary restriction field would be helpful and the significance of highly relevant previous literature that was not cited nor considered in the current manuscript. The authors should read Park et al., 2010 and other papers from that group that are relevant to dietary restriction, axenic dietary restriction, and cup-4. The difference between the previous work and this current manuscript should be discussed. Originality of work is an important criterion for PLOS One; "If a submitted study replicates or is very similar to previous work, authors must provide a sound scientific rationale for the submitted work and clearly reference and discuss the existing literature. Submissions that replicate or are derivative of existing work will likely be rejected if authors do not provide adequate justification."

We added additional literature to the introduction and discussed it in relation to our own results (lines 41-50 and 288 of the revised manuscript with mark-up). A more detailed reply to Reviewer 1 concerns can be found below.

Reviewer 2 suggests several experiments that could be done to address how much of the reduction in ADR-mediated lifespan extension observed upon coelomocyte ablation is accounted for by CUP-4. Previous work may help address this question, and while I agree that these suggestions by Reviewer 2 are interesting and important, they are not necessary in revising the current manuscript.

We thank you for this clear communication of revision expectations. We addressed all the proposed experiments of Reviewer 2 below and included their main concepts in the discussion of our revised manuscript.

**Reviewer 1 comments**

1. It is widely known that both dietary restriction (DR) and axenic culture can increase the lifespan of *C. elegans*. Axenic dietary restriction (ADR) would probably have the combined effect of both conditions. Therefore, it should be reasonable to discuss what is known in DR studies that are highly related to this research. It is also debatable whether the effect observed by the authors between fully fed (bacteria-fed) and ADR were caused by axenic, DR, a combination effect, or just simply different food source and nutrient content. A more fair comparison would be perhaps using killed bacteria.

We understand the reviewer’s concerns and hope we can resolve them through further clarification here and in the manuscript text. Axenic culture and axenic dietary restriction (ADR) are used as synonyms in the community, as axenic worms always have a DR phenotype. The exact reasons why axenic culture/ADR leads to lifespan extension are still unknown. However, it has already been shown that using killed bacteria does not have the same effect as ADR, so there must be something else at work besides the absence of live bacteria (lines 36-38 of the revised manuscript with mark-up). As it is currently unknown whether the slight lifespan extension seen by feeding dead bacteria might be part of the strong extension seen under ADR, it is currently inadvisable to use this as a control condition. Instead, the established FF condition is a wiser choice, and allows to compare with existing ADR literature (also relying on FF as reference).

2. A previous paper (Park et al., 2010) (and other papers from their group) suggest that cup-4 and coelomocytes are required for dietary restriction (DR) associated life extension. The paper should have been cited, and the difference between this manuscript and the paper should have been discussed. When discussing other genes in the paper, the authors also failed to discuss or mention highly relevant works that had been done in the DR field.

We agree that the Park group has already done important research on the topic of the coelomocytes and DR. This is why we already cited two (out of three) of their DR papers in our manuscript. In their 2013 paper, they show the importance of the coelomocytes in liquid and solid DR, and their 2017 paper does the same for bacterial dilution, peptone deprivation and food deprivation. It is certainly true that the Park, Link & Johnson 2010 paper laid the groundwork for these cited publications, and we agree with the reviewer it is worth discussing in our own manuscript. We therefore included this citation in our work (lines 41-50 and 288 of the revised manuscript with mark-up). Beyond *cup-4*, we believe that the relevant literature regarding immune genes in DR has been cited on the highlighted line 250 in the revised manuscript (with mark-up).

3. In Fig. 1B, since the fully fed is grown on solid mediums and the ADR in liquid, it is probably better to use separate plots to present the data as the solid and liquid medium is known to have a significant effect on the worm’s development.

We have modified the figure as requested by the reviewer.

**Reviewer 2 comments**

1. Does endogenous CUP-4 rescue completely rescue the reduction in ADR-mediated lifespan extension in coelomocyte-ablated animals?

We understand the motivation for the reviewer’s request, as it builds on an approach often used to understand combinatorial pathway or genetic logic. However, it has been shown through single cell sequencing (e.g., Hammarlund et al. 2018) and through a fluorescent reporter (this work) that *cup‑4* is solely expressed in the coelomocytes. Therefore, *cup-4* cannot be rescued in a coelomocyte-ablated animal, as the cell where it is expressed and exerts its function is absent, so the proposed experiment would unfortunately not work.

2. Do other components involved in coelomocyte endocytosis regulate ADR-mediated lifespan extension?

We agree that it would be very interesting to determine the FF and ADR lifespans of several other “coelomocyte uptake defective (*cup*)” mutants, to further strengthen the role of coelomocyte endocytosis in axenic longevity. However, there are a number of complications associated with the fact that *cup‑4* is the only one of these genes that is coelomocyte-specific, while other *cup* genes are more broadly expressed. We and others are looking into ways to deal with this for future exploration. In response to the reviewer’s question, we now included this notion in the revised manuscript (lines 285-287 of the revised manuscript with mark-up).

3. (a) The *dbl-1* mutant shows a reduction in ADR-mediated lifespan extension – does this effect persist in a lifespan assay performed without FuDR? (b) Are the effects of *dbl-1* and coelomocyte ablation (or *cup-4* loss-of-function) on the extended lifespan under ADR additive, i.e., do DBL-1 and coelomocyte endocytosis act independently to regulate lifespan under ADR? If so, then the paper can be about how coelomocyte endocytosis and innate immune pathways independently regulate lifespan upon ADR.

While these are certainly interesting questions, unfortunately, (a) one cannot completely avoid the use of FUdR in these experiments because ADR worms in liquid cannot be transferred to fresh medium to separate them from larvae. Although a lifespan experiment without FUdR would indeed be of great interest, we are not aware of any suitable longevity setup that avoids FUdR and is compatible with ADR (highlighted in lines 259-261 of the revised manuscript with mark-up). An alternative would be the use of sterile mutants, but this is undesirable as it not only is a strong confounding variable, but also is accompanied by practical issues (e.g., having to cross all mutant strains into the sterile background). (b) Due to this currently unavoidable interaction effect with FUdR, further experiments with *dbl-1* seem inadvisable at this time.

4. How does CUP-4 regulate lifespan extension in ADR? Does LIPL-5 in coelomocytes or intestinal fat content change upon ADR and do these involve CUP-4 or coelomocyte endocytosis?

At the moment, effectors of CUP-4 remain unknown. There is indeed a link between the lipase LIPL-5, the coelomocytes, and longevity by bacterial deprivation, another form of DR (Buis et al. 2019). As the focus of our work is the potential involvement of immunity, LIPL-5 was not a candidate in our study; nevertheless, it forms an interesting line of inquiry for future research. We would like to note that in stark contrast to the previous research communications by the Park lab (2013, 2017) as well as our own research, Buis et al. find an *increased* lifespan extension by DR upon coelomocyte ablation. One possibility for this difference may be due to their use of *E. coli* HT115 as a food source, as opposed to OP50 used by us and the Park team. Feeding on HT115 *vs* OP50 is known to have a significant effect on lifespan, fat content and *lipl-5* expression (Stuhr & Curran 2020). Moreover, some genes seem to be crucial to DR longevity when fed on one food source while being dispensable to it on another food source (Torzone et al. 2023). While all of this would need to be tested for LIPL-5, its role may thus be more related to the food source prior to DR. LIPL-5 therefore is certainly an interesting candidate to study how life history traits may influence differential effects of coelomocytes on DR-mediated longevity. As our results suggest that immune signalling cannot explain the importance of the coelomocytes in ADR, LIPL-5 and fat metabolism could form interesting alternative candidates for future research.

5. (minor) Does *cup-4* expression in wt background extend wt lifespan?

It could indeed be interesting to generate a *cup-4* overexpressor and test its lifespan, to determine whether increased endocytosis might further lengthen lifespan. We included this question in lines 280-282 of the revised manuscript (with mark-up).

6. I also suggest the following text and figure changes for clarity:
a. The authors have included p-values in Figure legends – they can consider also including them in the figures on top of the bar plots or next to the survival curves, at least for some of the comparisons.

We tried this, but unfortunately the figures became overcrowded and less clear, so we reverted this change back to the original. Since the other reviewer did not comment on this, we hope our current data representation will be considered the most clear by the majority of readers.

b. Page 2, line 36 – consider replacing ‘DAF-16a’ with ‘DAF-16’

One of the strengths of the new *daf-16* CRISPR knockout mutant is that it deletes all isoforms, while previous research truly was restricted to the DAF-16a isoform. Therefore, this is the most precise description of available information.

c. In Figure 2d, consider replacing ‘wt (backcrossed)’ with’ wt (KU25 outcrossed to N2)’

We changed the figure legend to “wt (N2 outcrossed to KU25)”, as it is the wild-type *pmk-1* gene in a KU25 background.

d. Figure S1 – typo: replace ‘oberved’ with ‘observed’

Done.

e. Page 10, lines 166-167; if the authors do not find a link between coelomocyte endocytosis and other immune pathways and instead discover that they act independently (see experiments suggested above) they can consider rephrasing these sentences to ‘Coelomocytes are immune cells which ‘phagocytose’ pseudocoelomic content. We wondered whether other immune pathways are involved in ADR-mediated longevity.

Addressed in point 3 above.

f. Page 9, line 151 – ‘localization’ instead of ‘localisation’

Done.

g. Page 10, line 183 – typo: change to ’slightly short-lived (p=1.9E-05) under ADR, but an interaction effect with FUdR is likely’

Done.

h. Page 13, line 241 – ‘hypothesized’ instead of ‘hypothesised’

Done.

i. Figure 1 – consider rearrange panels? The order of C and D are confusing.

Done.

j. For Figure S2, consider referring to Table S1.

Done.
